# Neural Correlates of Simulated Driving While Performing a Secondary Task: A Review

**DOI:** 10.3389/fpsyg.2019.01045

**Published:** 2019-05-09

**Authors:** Massimiliano Palmiero, Laura Piccardi, Maddalena Boccia, Francesca Baralla, Pierluigi Cordellieri, Roberto Sgalla, Umberto Guidoni, Anna Maria Giannini

**Affiliations:** ^1^Neuropsychology Unit, I.R.C.C.S. Fondazione Santa Lucia, Rome, Italy; ^2^Department of Life, Health and Environmental Sciences, University of L'Aquila, L'Aquila, Italy; ^3^Department of Medicine and Health Sciences ‘Vincenzo Tiberio’, University of Molise, Campobasso, Italy; ^4^Department of Psychology, University “Sapienza” of Rome, Rome, Italy; ^5^Ministry of Interior, Department of Public Security, Rome, Italy; ^6^ANIA Foundation, Rome, Italy

**Keywords:** distracted driving, language, audio, visual, attention, prevention

## Abstract

Distracted driving consists in performing a secondary task while driving, such as cell-phone conversation. Given the limited resources of the attentional system, engaging in a secondary task while driving increases the risk to have car accidents. The secondary task engagement while driving can depend on or be affected by different factors, including driver's individual characteristics, necessities, environmental conditions, and so forth. In the present work, the neuroimaging studies that investigated the brain areas involved in simulated driving during the execution of a secondary task (visual and overall auditory tasks) were reviewed in light of driving settings. In general, although there are also differences in decrease and increase brain activations across studies, due to the varieties of paradigms used (simulators, secondary tasks and neuroimaging techniques), the dual-task condition (simulated driving plus secondary task), as compared to the simulated driving-alone condition, was generally found to yield a significant shift in activations from occipital to fronto-parietal brain regions. These findings show that when a secondary task is added during driving the neural system redirects attentional resources away from visual processing, increasing the possibility of incorrect, dangerous or risky behavioral responses. The shift of the attentional resources can occur even if driving behavior is not explicitly affected. Limits of the neuroimaging studies reviewed and future research directions, including the need to explore the role of personality factors in the modulation of the neural programs while engaging distracted driving, are briefly discussed.

## Introduction

Driving is a complex activity that involves several mental cognitive processes requiring the coordination of different abilities, such as visuo-spatial attention, visuo-motor, and auditory skills (Graydon et al., 2004). In particular, the driving task is based on continuous adjustments and reallocation of attention, that can be affected by different sources of distraction. In the real-world, distraction may be due to different factors that generally lead drivers' eyes or mind off the road, such as traffic density, speed, driver psychophysiological conditions (e.g., sleepiness, mood), type of road, weather and so forth (Oron-Gilad and Ronen, 2007; McGehee, 2014). In addition, despite the complexity of the driving task, drivers usually engage in secondary tasks for different reasons, including the attempt to make the time spent on the roadway more productive (Reschovsky, 2004). These secondary tasks include more traditional activities, such as talking to passengers, listening to the radio, eating, drinking, lighting a cigarette, applying makeup (e.g., Stutts et al., 2003), as well as cell-phone related activities, that is having conversations by mobile, surfing the internet, sending and receiving e-mails, or faxes and texting. Thus, the secondary task generally involves removing cognitive resources off the immediate driving task and sometimes also removing driver's eyes off the road or hands off the wheel (National Highway Traffic and Safety Administration (NHTSA), 2015). It generally increases the working memory load and is not appropriate for maintaining alertness (Oron-Gilad et al., 2008).

In this vein, activities performed using new technologies (e.g., Smartphone) are more distracting because they are more cognitively engaging and are performed over longer periods of time (Strayer et al., 2006). According to different experts and studies the use of cellular phones while driving enormously contributes to collisions between motor vehicles (e.g., Violanti and Marshall, 1996). For example, holding a complex conversation by cell-phone also affects driving performance (e.g., McKnight and McKnight, 1993). Even processing of a single, verbally presented word was found to negatively affect driver braking response (Rossi et al., 2012). For these reasons, different countries (e.g., Brazil, Israel, Australia, Italy) prohibited using smartphones/cellular phones (hand held) while driving. However, Dingus et al. (2011) revealed that eating or reaching for objects in the vehicle while driving were also associated with high increased odds of having a motor vehicle collision or near-crash.

Given these implications, the understanding of factors that lead drivers to get engaged in distracted driving (e.g., driver's individual characteristics, driving experience, necessities, environmental conditions) is extremely important to better implement strategies aimed at preventing fatal accidents. For example, as concerns personality traits, Parr et al. (2016) revealed that in teens high openness and conscientiousness predicted the secondary task engagement while driving, such as texting frequency and interacting with a phone, whereas low agreeableness predicted lesser texting frequency and interacting with a phone; in older adults, extraversion predicted talking on and interacting with a phone. However, the engagement in secondary tasks requiring drivers to look away from the road ahead is generally more risky for novice than expert drivers (Klauer et al., 2014). Interestingly, the individual attitude toward daydreaming/mind wandering can also be risky while driving, especially under monotonous driving circumstances. In such cases, the engagement in a secondary task can be the lesser of the two evils, reducing the chance of mind wandering to intrude the primary activity, when the driving setting is monotonous (Nijboer et al., 2016).

Although the study of the role of factors related to the drivers' individual characteristics or environmental conditions in distracted driving appears to be crucial, research in the field is scarce. In addition, the way in which such factors affect neural correlates of distracted driving is even more neglected by the experimental research. In the last two decades the application of neuroimaging techniques has been used in association with simulated driving and multitasking using different methodologies, but no study has considered the modulation of personality factors. Only some studies considered to some extent the environmental conditions associated to the secondary task engagement, mainly using simulation contexts. In this direction, more insights might be gained moving from the general driving settings. Therefore, in the present paper the brain systems that are mostly involved in distracted driving are explored in light of the driving setting, that is, on the basis of the type of the primary driving task (that also relies on the driving scene) combined with the secondary task. More specifically, here we aimed at understanding whether brain activations associated with driving decrease when a secondary task is added, in spite of driving and distracting tasks draw on different cortical areas. This would allow to understand if during distracted driving changes in brain activations occur also in absence of behavioral changes.

## Inclusion Criteria for Papers

The literature was reviewed using a systematic method. PubMed, Science Direct and Web of Science were used as databases with the following strings “driving and multitasking” or “distracted driving” plus one of the following words: “neuroimaging,” “fMRI,” “MEG”. Sixteen papers were found. The a priori inclusion criteria were seven: (1) neuroimaging studies had to be based on fMRI and Magnetoencephalography (MEG) techniques. These studies were preferred because of their relative satisfactory spatial and temporal resolution; on the contrary, Positron Emission Tomography (PET), Single Photon Emission Computed Tomography (SPECT) were not included due to their very low temporal resolution, whereas Electroencephalography (EEG) and Near-infrared Spectroscopy (NIRS) studies were not included due to their very low spatial resolution. (2) Studies had to include at least one condition in which participants were specifically instructed to drive and simultaneously to perform on a secondary task (e.g., visual, auditory in nature); thus, neuroimaging studies focused on driving only were excluded. (3) All participants in the studies had to be healthy adults. (4) All neuroimaging studies had to include a control condition (baseline), that is an appropriate matched control condition (e.g., driving + secondary task vs. driving only), to exclude all the activations that were not directly connected to distracted driving. (5) Only group studies were included, that is studies with at least five participants. (6) There could be no pharmacological manipulation. (7) Only peer-reviewed original articles published in established scientific journals were included; conference papers were excluded.

Using these criteria, we selected 11 papers, 9 fMRI, and 2 MEG papers (see Table 1).

**Table 1:** Neuroimaging studies included in the present review.

**Study**	***N***	**Mean age**	**Age range**	**DE**	**SD**	**Secondary task**	**Behavioral results**	**Contrast condition and main neuroimaging results**
fMRI (Graydon et al., 2004)	6–3F	–	22–28	.	Real world highway presented in a fronto-parallel viewing perspective	Visual event detection task (CEDR—central event detection response, to red colored stimuli	SD + CEDR Task = 611 ms	SD + CEDR vs. SD
							CEDR Task = 550 ms	Activation of Fronto-parietal regions
							*t* = −2.68, *p* < 0.05	
fMRI (Just et al., 2008)	29–14F	–	18–25	.	Steering a vehicle along a curving virtual road using a trackball or mouse in the right hand	Listening and answering to true/false pre-determined questions	SD + Audio Task = 92% (ACC)	SD + Audio Task vs. SD
							Mean road-maintenance errors:	Activation of the bilateral temporal language areas, left inferior frontal gyrus;
							*SD* = 8.7 (9.7)	Decrease of activation in spatial brain areas
							SD + Listening = 12.8 (11.6)	
							*t*_(28)_ = 2.22, *p* < 0.05	
							The mean root mean squared deviation from the ideal path:	
							SD + Listening = 2.64 (0.56)	
							*SD* = 2.48 (0.51)	
							*t*_(28)_ = 2.79, *p* < 0.01	
MEG Bowyer et al., 2009	28–17F 19 MEG	36.5 (13.8)	18–65	–	Similar to Bowyer et al. (2007)	Listening and answering to pre-determined questions – 1 question = short conversation; multiple questions = long conversation	Results 19 subjects in MEG	SD + Long Conversation Task vs. SD
							SD + Long Conversation Task:	Decrease of activation in the visual cortex and in the right superior parietal region
							RT = 1,043 ms-SE = 65 ms;	
							SD = RT = 944 ms-SE = 48 ms	
fMRI (Hsieh et al., 2009)	10–6F	36.9	19–61	–	Similar to Bowyer et al. (2007, 2009)	Listening and answering to pre-determined questions in order to carry on short and long conversations	Results in fMRI:	SD + Long Conversation vs. SD
							SD + Long Conversation:	Activations of language areas (e.g., Wernicke's and Broca's areas) and fronto-parietal areas
							RT = 770 ms;	
							SD (no conversation) = RT = 726 ms	
MEG (Fort et al., 2010)	13 M	25.4 (2.1)	21–28	≥3 year	Driving on single or dual roadways, following traffic light rules, and direction signs, with little traffic and few pedestrians on the roads	Listening to broadcast and answering to 3 pre-determined questions (for half of participants)	SD (traffic lights) + Audio Task	SD + Audio Task vs. SD
							RT = 430 ms	In both conditions, with traffic light or arrows, sensory visual areas and right fronto-parietal network were activated
							SD (traffic lights): RT = 399 ms	With traffic lights: decreases of brain activity in primary visual areas, dorsolateral prefrontal cortex, and right temporo-parietal junction; increase of activation in the posterior parietal cortex. With arrows: decreases of brain activity in occipital visual areas, frontal areas, including the premotor area and left posterior parietal area; increase of activity in the frontopolar cortex
							*F* = 8.167; *p* = 0.013	
							SD (arrows) + Audio Task	
							RT = 875 ms	
							SD (arrows): RT = 890 ms	
							*F* = 2.301; *p* = 0.153	
fMRI (Uchiyama et al., 2012)	18–8F	27.7 (4.3)	–	–	Following a car at the distance of 5 m using a joystick with the right hand. No intersections, other vehicles, or obstacles were included	(1) Sentence comprehension: judge whether the subject of the verb corresponded to the person in the paired words; (2) tone discrimination: judge whether the beep tone in the response phase was high or low	Sentence comprehension accuracy:	SD + Audio Task vs. SD
							SD + Audio Task: 86.2%	Decrease of activations in the medial prefrontal cortex and left superior occipital gyrus
							Audio Task: 88.9% (*SD* = 12.23)	Car-following performance showed positive correlation with brain activity in the bilateral lateral occipital complex and the right inferior parietal lobule
							*t*_(17)_ = 0.90, *p* = 0.381	
							Sentence comprehension RT:	
							SD + Audio Task: 1,668 ms (320)	
							Audio Task: 1,841 ms (300)	
							*t*_(17)_ = 2.77, *p* < 0.05	
							Car-following performance worse during SD + Audio Task than during SD alone	
fMRI (Schweizer et al., 2013)	16–7F	25.8 (1.5)	20–30	7.4 (2.5)	Straight driving, turning left or right at intersection with or without incoming traffic using steering wheel and pedals	Listening and answering to pre-determined questions	SD (Straight Driving) + Audio Task	SD + Audio Task vs. SD
							Speed: 58.69 (2.34)	Shift in activation from the posterior to the anterior brain during the dual-task condition
							SD (Straight Driving) – Speed: 58.57 (3.36)	
							SD (Left turn traffic) + Audio Task – Speed: 28.98 (3.76)	
							SD (Left turn) – Speed: 26.79 (5.17)	
							SD (Left turn traffic) – Speed: 29.35 (4.26)	
fMRI (Chung et al., 2014)	16 M	26.6 (2.1)	–	2.7 (1.5)	Using the wheel and pedals to drive at a constant speed (110 km/h) on a straight road with very few distracting elements, without changing lanes	Listening and answering to questions regarding double-digit carry-over calculation with sums <100	–	SD + Calculation Task vs. SD
								Activations of cingulate gyrus and sub-lobar region
								Decrease of activation of regions associated with spatial processing, movement planning and execution
fMRI (Al-Hashimi et al., 2015)	31–14F	38.4 (6.3)	30–40	–	Keeping the car within a target box. Right and left turns, and inclining and declining hills formed the tracks, varying from mild to severe	Discrimination sign task (green circles −33% frequency—with a right button press)	Mean accuracy no significant differences	SD + Discrimination Sign Task vs. SD
							SD + Discrimination Sign Task: 513.9 ms	Activation of the right superior parietal lobule
							*SD*: 530.7 ms	
							*t*_(30)_ = 3.77, *p* = 0.00042	
fMRI (Sasai et al., 2016)	13 M	–	22–34	–	Minimizing the deviation from the simulated track centerline	GPS instructions (integrated task) or radio show (split task)	No significant difference in any measures between the integrated and split task conditions	Reduced multivariate functional connectivity during the split task between driving and listening network; higher integration of information content of driving and listening networks during the integrated task
fMRI (Choi et al., 2017)	15 M	26 (1.4)	–	2.5 (1.6)	Using the wheel and pedals to drive at a constant speed (80 km/h) on a straight road with very few distracting elements, without changing lanes	Similar to Chung et al. (2014)	Accuracy for SD + Calculation Task	SD + Calculation Task vs. SD
							78.5 ± 11.7%;	Inferior frontal gyrus and the superior temporal gyrus enhanced activation
							For Calculation Task: 84.8 ± 10.9%	
							*t*-test (PASW Statistics 18), *p* = 0.196	

*DE, driving experience; SD, simulated driving*.

## Driving Settings

In some studies the driving setting consisted in straight driving (Sasai et al., 2016), also on real world highways (Graydon et al., 2004; Bowyer et al., 2009; Hsieh et al., 2009). In other studies it consisted in driving on computerized roads at constant speed (Chung et al., 2014; Choi et al., 2017), following a car at the distance of 5 m (Uchiyama et al., 2012), or even following traffic light rules, and direction signs (Fort et al., 2010), including left and right turns, from simple (Just et al., 2008) to more complex driving scenes (Schweizer et al., 2013; Al-Hashimi et al., 2015). The settings of simulated driving were implemented using specific devices, such as, a trackball or mouse (Just et al., 2008), a joystick (Uchiyama et al., 2012), a game controller (Al-Hashimi et al., 2015), a steering wheel and foot pedals to control the accelerator and brake (Schweizer et al., 2013; Chung et al., 2014; Choi et al., 2017), or more sophisticated simulators (e.g., wheel, turning indicator, accelerator and brake pedal) (Fort et al., 2010). The type of simulator device was not specified in Sasai et al. (2016). In some studies only driving videos were presented, that is participants were instructed to watch and actively attend these videos without using a wheel or any other specific device (Graydon et al., 2004; Bowyer et al., 2009; Hsieh et al., 2009).

## Secondary Tasks

With the exception of Sasai et al. (2016), who presented a radio show (with no questions to be answered), the most of studies used auditory distracting tasks based on listening and answering to questions. In general, questions were presented through headphones (e.g., Just et al., 2008; Bowyer et al., 2009; Hsieh et al., 2009; Uchiyama et al., 2012; Schweizer et al., 2013), but also by radio broadcast (Fort et al., 2010) and using an audio system attached to the MR-compatible driving simulator (Chung et al., 2014; Choi et al., 2017). Different types of pre-determined questions were used: true/false questions, such as “A triangle has four sides?” (e.g., Just et al., 2008; Schweizer et al., 2013); questions requiring to answer whether the subject of the verb corresponded to the person in the paired words (Uchiyama et al., 2012); questions about double-digit carry-over calculation with sums <100 (Chung et al., 2014; Choi et al., 2017). Answering the pre-determined questions required to press true/false buttons or verbalize the response carrying-over calculations. In addition, open questions were also used, such as “…Do you have time to talk now?” or “What is your address?” (e.g., Bowyer et al., 2009; Hsieh et al., 2009; Fort et al., 2010), which were aimed at simulating short (1 question) and long (multiple questions) conversations (e.g., Bowyer et al., 2009; Hsieh et al., 2009). Participants were asked to covertly verbalize their responses. Only two studies used visual distracting tasks based on discrimination of signs, such as detecting red stimuli (Graydon et al., 2004) or green circles among other colored geometrical stimuli (Al-Hashimi et al., 2015) presented on the driving screen.

## Neural Correlates

In the study conducted by Fort et al. (2010) following traffic lights rules while listening and answering to ordinary open questions (dual task condition) yielded to decreased activations in the dorsolateral prefrontal cortex, the right temporo-parietal junction and in the primary visual areas, compared to the simulated driving-alone condition (single task). In addition, following direction signs during driving produced reductions in activations in the visual areas and in premotor area compared to the single task condition. On the contrary, increased activations were found in the left posterior parietal cortex both while following traffic lights rules and direction signs as compared to the single task condition.

In Uchiyama et al.'s (2012) study, following a car while answering questions about grammatical problems produced decreased activations in the medial prefrontal cortex and the left superior occipital gyrus as compared with the simulated driving-alone condition; instead, increased activations were found in the middle frontal gyrus. Interestingly, in this study the right inferior parietal lobe and the bilateral lateral occipital complex were found to correlate positively to the car-following performance during the dual-task, with decreased activation associated with worse performance.

Driving at constant speed while responding to questions about calculation problems yielded decreased activation in the left middle frontal gyrus (Chung et al., 2014; Choi et al., 2017), the middle occipital gyrus and the right superior parietal lobe (Choi et al., 2017), the right inferior parietal lobe, the supramarginal gyrus and the cuneus (Chung et al., 2014) as compared with the simulated driving-alone condition. In addition, increased activations were found in the orbitofrontal cortex, the bilateral lateral prefrontal cortex, the frontal eye field regions, the anterior and the posterior cingulate gyri, the lentiform and the caudate nuclei (Chung et al., 2014; Choi et al., 2017), inferior frontal gyrus and right superior temporal lobe (Choi et al., 2017).

Driving on computerized roads with left and right turns while responding to true-false questions damped activations in the bilateral superior parietal lobe, the bilateral intraparietal sulci, the bilateral superior extrastriate occipital cortex (Just et al., 2008) and the occipital visual regions (Schweizer et al., 2013) as compared with the simulated driving-alone condition. In addition, the bilateral temporal lobe, the left inferior frontal regions, the right supplementary motor area (Just et al., 2008), and the bilateral anterior brain areas, especially the dorsolateral prefrontal cortex and the frontal polar region (Schweizer et al., 2013) were found activated during dual-task as compared with driving-alone condition.

Watching and actively attending driving scenes while answering to open questions yielded decreased activations in the right superior parietal lobe and in the visual areas (Bowyer et al., 2009) as compared with the simulated drivingalone condition; on the contrary, brain activity in language-specific areas was found enhanced (Bowyer et al., 2009). Increased activations were confirmed in language specific areas (i.e., Broca and Wernicke's areas) extending also to the orbitofrontal cortex, the bilateral lateral prefrontal cortex, the frontal eye fields, the supplementary motor cortex, the anterior and posterior cingulate gyrus, the inferior frontal gyrus, the middle frontal gyrus, the right superior parietal lobule, the right intraparietal sulcus, the right precuneus, and the cuneus (Hsieh et al., 2009).

Watching and actively attending driving videos while detecting visual stimuli yielded increased activations in the superior parietal lobule, the bilateral precentral gyrus, the bilateral superior frontal gyrus, the middle frontal gyrus, the frontal eye fields, the cingulate cortex, the inferior parietal lobule, and the cerebellum as compared with the simulated driving-alone condition (Graydon et al., 2004). Driving on complex computerized roads while detecting visual stimuli confirmed the increased activation of the right superior parietal lobule, compared to the simulated driving-alone condition (Al-Hashimi et al., 2015).

## Discussion

In the present review the neural correlates of distracted driving were explored on the basis of the type of the primary driving task combined with the secondary distracting task, in order to gain insights on some of the environmental characteristics that can cause unsafe driving. The aim was to clarify if brain activations associated with driving decrease when a secondary task is added, even though the two tasks rely on different cortical areas, in order to gain insight on changes of neural activities even when driving behavior is not explicitly affected. Taken together the neuroimaging results showed, with some exceptions, that during the simulated distracted driving a significant shift in activations occurs from the posterior to the anterior cerebral regions. Actually, the occipital areas were less involved during simulated distracted driving compared to the simulated driving-alone condition; also, greater recruitment of frontal areas occurs during simulated distracted driving (e.g., Just et al., 2008; Bowyer et al., 2009; Uchiyama et al., 2012; Schweizer et al., 2013; Choi et al., 2017). This general shift seems to be consistent across studies, regardless the type of questions posed (i.e., closed or open) and the type of response given (i.e., button press or vocal), and sometimes occurs even in absence of clear change in driving behavior, such as while driving following direction signs (e.g., Fort et al., 2010) or during straight driving (e.g., Schweizer et al., 2013), with the implication that the risk of having car accidents increases anyway.

In detail, in some studies that involve language-based secondary tasks, the shift of activation is more consistent toward the fronto-temporal language areas (e.g., Bowyer et al., 2009; Hsieh et al., 2009; Choi et al., 2017), especially using open questions and vocal responses. According to Liu et al. (2012) the prefrontal cortex is involved in the preparation processing before the turning behavior regardless of the cognitive load. However, these authors also showed an increasing pattern of prefrontal activation from the pre- to the post-turning throughout the actual-turning period when participants had to follow verbal instructions regarding turns (extrinsically driven cognitive load), as compared with driving using a memorized map (intrinsically driven cognitive load). Thus, the greater involvement of frontal areas during distracted driving might reflect a possible competition for limited resources and attentional reallocation (Wickens, 2008). In particular, the prefrontal cortex plays a key role on goal-directed stimulus selection and response as a top–down attention control, coordination of temporal order for task interference and mapping concurrent sensory information in terms of motor behavior (e.g., Adcock et al., 2000; Stelzel et al., 2006).

This means that visual attention is sacrificed while people are engaged in distracted driving, even though there are no significant changes in some indices of driving behavior. This view is supported by the evidence that the frontal eye field (e.g., Graydon et al., 2004; Hsieh et al., 2009; Choi et al., 2017) mediates visual attention for visual fields, and visual attention influence for the sensitivity of extrastriate visual cortex (Ruff et al., 2006; Silvanto et al., 2006). In other words, a secondary task decreases foveal attention to visual information while driving, even though fixation is not affected (Strayer et al., 2003). In this direction, the “inattention blindness” phenomenon (Simons and Chabris, 1999), that is the individual's failure to see unexpected and often salient stimuli that are in plain sight, has to be considered. Indeed, the inattentional blindness occurs when one is simply attending to something else, such as happens during distracted driving, and can relate directly to specific road accidents, especially among novice drivers.

Different studies also found increased activation in the right superior parietal lobe during distracted driving, when the secondary task was visual (e.g., Graydon et al., 2004; Al-Hashimi et al., 2015) or auditory (Hsieh et al., 2009) in nature. This area is also involved in visual attention and awareness, as well as into the modulation of the neural activity in extrastriate visual cortex (Beck et al., 2006) and shifts in attention (Vandenberghe et al., 2001). Specifically, this parietal area may reflect attentional engagement or cognitive control that subserve the switch between the primary and secondary tasks (Shapiro et al., 1997; Dux and Marois, 2009). However, other studies based on auditory secondary tasks found that the activations of the right superior parietal region decreased in the dual-task condition as compared to the simulated driving-alone condition (e.g., Just et al., 2008; Bowyer et al., 2009; Choi et al., 2017). This might suggest that the activation of the right superior parietal lobe seems to be sensitive to the type of the secondary task. However, the extent to which this area is really crucial for attentional engagement should be clarified by future studies.

Interestingly, a shift of activation seems to occur more specifically in terms of motor areas. Indeed, on the one hand, different regions of the motor systems were found activated (e.g., Graydon et al., 2004; Choi et al., 2017); amongst others, the supplementary motor cortex, that contributes to different cognitive functions, such as the coordination of temporal sequences of actions (Lee and Quessy, 2003) and bimanual coordination (Serrien et al., 2002), was recruited using both simple computerized and more complex real-world driving scenes (e.g., Just et al., 2008; Hsieh et al., 2009). On the other hand, the activation of the middle frontal gyrus, which is involved in movement planning and execution, decreased during driving at constant speed on computerized roads while performing double-digit carry-over calculations (e.g., Chung et al., 2014). In other words, during the distracting driving there are decreased activations of the motor brain areas directly associated to driving, with detrimental effects on vigilance, coordination, preparatory components and timing of motor responses, and increased activation of those brain areas that mediate error monitoring and unnecessary movements control. This pattern of results seems to occur regardless of the type of the secondary task and questions posed.

These preliminary neuroimaging results show that distracted driving yields a reallocation of attentional resources at neural level, with the possibility that incorrect or dangerous behavioral responses are adopted while driving. Attentional resources are re-directed away from visual or motor processing when a secondary task is performed during driving, and some of the neural programs going on can cause car accidents, even if driving behavior is not explicitly affected. From this picture it seems that attention and arousal at neural level are affected earlier than observed behavioral measures. This new evidence poses the issue of the extent to which distracted driving is compatible with effective distributed attention resources. In this direction, Sasai et al. (2016) found that when participants were engaged in simulated driving while listening to radio show (split task) the functional connectivity between the two hemispheres decreased, giving rise to “functional split brain” as normally occurs in patients with a Corpus callosotomy. On the contrary, when participants listened to Global Position System (GPS) instructions while driving (integrated task), the connectivity between the two hemispheres increased. Well, although from this study the decrease of functional connectivity from high to low information integration is compatible with the split in consciousness, that is with two independent functional streams, the possibility that performing a secondary task absorbs attentional resources primarily at neural level, making driving unconscious, as on autopilot, with obvious consequences for safety, should be considered.

In conclusion, from this review appears that more work is necessary to clarify the extent to which the factors related to driving settings affect neural correlates of distracted driving. The number of studies available is scarce and the substantial differences due to the varieties of paradigms used (simulators, secondary tasks and neuroimaging techniques) make difficult to draw definitive conclusions, even though it is possible to get some indications for future research. The most important implication of this review is that when a secondary task is added during driving, the neural system re-directs attentional resources away from the primary task, increasing the possibility for car accidents. In addition, even though some studies have not collected RTs and even miss rates for the tracking tasks (e.g., Graydon et al., 2004; Just et al., 2008; Uchiyama et al., 2012; Choi et al., 2017), making difficult to get a reliable effect of the secondary task on driving at both neural and behavioral levels, it appears that distracted driving yields to neural programs that reveal in advance possible behavioral consequences. This can represent a new research line in the understanding of human driving behavior, which usually appears to be highly automated, but also highly modifiable in terms of neural programs. The in-depth analysis of such an issue can help to implement learning driving programs. In this vein, since some behavioral studies revealed that there is a null effect on lane keeping variation with increased cognitive load (for a meta-analysis see Horrey and Wickens, 2006), the neuroimaging studies reviewed in the present paper should be supported by studies aimed at collecting on-road data. That is, the activations during simulated driving not necessarily reflect the exact pattern of activations that would occur in real-world driving conditions. Thus, decreases of activations in critical visuo-spatial areas (e.g., occipital regions) or the absorption of attentional resources in the dual-task condition might be even stronger in real multitasking driving. Future neuroimaging studies should better highlight the relationships between more fine-graded behavioral indexes and neural distracted driving correlates.

Finally, critical variables that might affect neural correlates during distracted driving, such as the type of the distracting task (e.g., passenger conversation and cell phone conversation) should be compared in order to understand the differential impact on neural mechanisms underpinning attentional processes. Despite, some study found no difference between remote (cell phone) and in-person (passenger) conversation in terms of attention performance (Amado and Ulupinar, 2005), it is not possible to exclude changes at neural level according to the type of conversation. Personality (Parr et al., 2016), driving styles (e.g., Lucidi et al., 2010; Giannini et al., 2013; Pierro et al., 2013; Sagberg et al., 2015), gender (Irwin et al., 2011; Cordellieri et al., 2016), age (Thompson et al., 2012; Cordellieri et al., 2016) of the driver, and the amount of driving experience (Klauer et al., 2014) must be also considered to get more reliable results in terms of neural correlates. Yet, emotional factors should have also accounted for. For example, using the static load paradigm while carrying out an emotional conversation task (different questions were presented using a neutral or angry speech tone), Hsieh et al. (2010) revealed by a congress paper that the angry emotional tone enhanced the right fronto-parietal networks and yielded desynchronizing or dampening of the left frontal activity as compared to neural emotional tone. Future research should explore the neural correlates involved in distracted driving considering different mediating factors. This integrated approach will definitively improve the prevention of unsafe driving.

## Author Contributions

MP: participated to the bibliography search and to writing. LP, MB, and AG: participated to writing and to define the theoretical implications. FB: participated to writing. PC: participated to bibliography search. RS and UG: participated to define the theoretical implications.

### Conflict of Interest Statement

The authors declare that the research was conducted in the absence of any commercial or financial relationships that could be construed as a potential conflict of interest.
